# Interpersonal violence and recurrent headache among adolescents with a history of psychiatric problems

**DOI:** 10.1186/s12991-023-00432-7

**Published:** 2023-01-24

**Authors:** Hanne Klæboe Greger, Sara Konstanse Kristianslund, Synne Øien Stensland

**Affiliations:** 1 Regional Centre for Child and Youth Mental Health and Child Welfare, Institute of Mental Health, Norwegian University of Science and Technology, Norway and St.Olavs Hospital, Trondheim, Norway; 2 Norwegian Institute of Public Health, Oslo, Norway; 3 Norwegian Centre for Violence and Traumatic Stress Studies, Oslo, Norway

**Keywords:** Interpersonal violence, Headache, Child, Adolescent psychiatry

## Abstract

**Background:**

Interpersonal violence (IPV) is found to be associated with mental health problems and pain disorders such as headache among children and adolescents. It is well-known that adolescents in need of mental health services have experienced IPV more often than adolescents in the general population. However, there has not been much focus on pain conditions in child and adolescent psychiatric populations.

**Methods:**

Data from the current study are based on a 3-year follow-up of the CAP-survey, which is a study of adolescents in the child and adolescent psychiatric unit population of St. Olavs Hospital (Trondheim University Hospital). The baseline study was conducted between 2009 and 2011, with 717 participants between 13 and 18 years. All participants were enrolled, or newly referred to the child and adolescent psychiatric clinic. At follow-up, 570 participants completed questionnaire, and 550 completed a diagnostic interview. The participants were aged 16–21 years (mean age 18.6 years).

**Results:**

A third of the adolescents reported frequent headaches (weekly or daily). Adolescents with more severe mental problems were more likely to experience frequent headaches. Adolescents exposed to unpleasant sexual acts or bullying, reported more frequent headaches than non-exposed participants. Participants exposed to three or more types of IPV seemed to be at particularly high risk of experiencing frequent headache.

**Conclusions:**

Both experiences of interpersonal violence and headache are common in this clinical psychiatric population. Clinicians should assess for headache disorders in addition to psychiatric and trauma assessment and provide need-based treatment to enhance chance of recovery among adolescents in mental health services.

## Background

Children and adolescents commonly experience mental health problems at some point during childhood. If severity or persistence of symptoms threatens everyday function and development, the child or adolescent will often be referred to child- and adolescent psychiatric clinics (CAP) to provide need-based help for recovery. Interventions are largely put in place based on diagnoses resulting from a thorough assessment of psychological symptomatology and related biopsychosocial risk and resilience factors. Despite a broad-based approach in child- and adolescent psychiatric assessments, there is a possibility of important shortcoming. In particular findings from past years research indicates children and adolescents in the mental health services commonly experience chronic pain and pain-related disability [1]. Traditionally such comorbidity has been left unaccounted for. Headache is one of the most common pain conditions in adolescents [2, 3]. Experiencing pain in adolescence can have a large impact on daily life functioning, as it can result in problems with sleep, in school and social function [2, 3]. Children with frequent headaches are at risk of recurrent headaches in adulthood as well as for having physical and psychiatric symptoms, compared with children without frequent headaches [4]. If not accounted for, there is a chance that uncovered pain could hamper children and adolescents’ ability to adhere to treatment plans or intervention strategies and thereby stand in the way for healthy recovery from their mental health problems [5]. Furthermore, research over the past decades has also revealed that if asked many children and adolescents in the mental health services report exposure to sexual abuse, physical or psychological violence, including bullying and child maltreatment [6, 7]. Exposure to interpersonal violence (IPV) seem to pose a particularly malevolent risk factor for both conditions. Yet, only about half of children and adolescents are asked about their traumatic experiences upon referral to CAP [8]. Currently, the level of unmet needs is high among children and adolescents with severe mental health problems. Increased knowledge of prevalence of common somatic symptomatology, such as frequent headaches, exposure to interpersonal violence and the relationship between the two among children and adolescents in the mental health services could help estimate the scope of the challenge ahead.

Mental problems and headaches both increase in prevalence with the onset of puberty, when headaches and psychological distress become increasingly prevalent particularly among girls. The etiology of mental problems and headaches in childhood and adolescence is multifaceted, can be related or relate to separate causal mechanisms [9]. IPV, such as physical violence, sexual abuse and bullying, can have huge consequences for exposed children and adolescents. Recent research has shown an association between exposure to IPV and a broad spectrum of mental and physical health problems, and psychosocial problems [10–18]. Children and adolescents are especially prone to the onset of health problems due to ongoing psychological, physical, and social development. Research results show that exposure to IPV at this stage of life can affect the persons self-confidence, view on self-worth and trust in other people [19].

Childhood experiences of IPV is a huge global problem. The World Health Organization (WHO) estimate that one out of two children in the age 2–17 years, experience some sort of violence each year [20]. In a study of Norwegian adolescents aged 12–16 years, about 5% reported experiences of severe physical violence, and about 5% reported experiences of sexual abuse from an adult [21].

In particular, frequent headaches are known as one of the most common causes of considerable functional impairment in children and adolescents [22]. The headaches of those exposed to IPV appear to be more severe than the headaches of the non-exposed, as exposure to IPV has been shown to increase the risk of frequent and intense headache [23]. The risk of chronic migraine in 1348 patients diagnosed with migraine at 11 headache centers in USA and Canada (88% women, mean age 41 years) was significantly associated with emotional abuse, physical abuse, physical neglect, and emotional neglect, although not with sexual abuse. The association between chronic migraine and emotional abuse was particularly strong [24].

A large amount of the existing research on the effects of adversities in childhood on health outcomes later in life show that there is an association between being exposed to IPV and suffering from headaches [13, 23, 25, 26]. In the Young-HUNT3 study on Norwegian adolescents in the general population, the association between IPV and headaches recurring monthly, weekly, or daily was significant and strong, and particularly strong for weekly and more frequent headaches. The prevalence of recurrent headache increased the more types of IPV the adolescents had been exposed to [18]. Comparable results with increased risk of headache for a higher number of IPV categories experienced were also found in adults in the ACE study [27], the Canadian Community Health Survey—Mental Health study [28], and the Add Health study [29].

The current study includes a clinical population of adolescents admitted to a child and adolescent psychiatric clinic. CAP units assess and treat children and adolescents with a range of mental health problems, such as neuropsychiatric disorders, affective disorders, and behavioral problems. Many of these adolescents have experienced IPV [7]. Previous results from the current study has shown that three out of four adolescents still had a psychiatric disorder 3 years after inclusion, and that chronic pain, smoking and trying illicit drugs were associated with persisting psychiatric disorders [30].

There has not been much focus on pain conditions in psychiatric clinical populations, and to the best of our knowledge, the association between IPV and recurrent headache has not been investigated to a large extent before. Being able to identify adolescents with an increased risk of headache is of great importance, as it would aid us in providing better healthcare services for these patients. The aims of this study are, therefore, to expand present knowledge of the prevalence of frequent headaches and its relationship to exposure to IPV among adolescents with a history of mental health problems, and more specifically we aim to: (i) estimate the prevalence of frequent headaches among adolescents with a history of mental health problems (ii) assess whether frequent headaches are more common among adolescents with more severe mental health problems, fulfilling diagnostic criteria of mental disorders and (iii) explore whether exposure to IPV is related to increased risk of frequent headaches among adolescents with a history of mental health problems.

## Methods

### Population and procedure

This study was based on data from The Health Survey in the Department of Child and Adolescent Psychiatry (CAP survey), which is a study of adolescents in the child and adolescent psychiatric unit population of St. Olavs Hospital (Trondheim University Hospital). The CAP survey is a prospective, longitudinal study, investigating the mental and physical health of the participants from adolescence into adulthood.

The baseline study (T1) was conducted between 2009 and 2011, with 717 participants between 13 and 18 years. All patients who were enrolled or newly referred at the CAP clinic in the study period received oral and written invitations at their first attendance, except for emergency patients who were invited once they entered a stable phase. The inclusion criteria were that they were aged between 13 and 18 years and had at least one personal attendance at the clinic in between February 15, 2009 and February 15, 2011. Major difficulties in answering the questionnaire due to psychiatric state, cognitive function, visual impairment or lack of sufficient language skills served as exclusion criteria, which excluded 289 adolescents. 95 were lost to registration (missing). Therefore, 1648 (81.1%) were invited to participate, and out of these, a total of 717 (43.5%) participated in the CAP survey at T1; 393 (54.8%) girls and 324 (45.2%) boys.

The follow-up study, T2, was conducted 3 years later (2012–2014), when the participants were between 16 and 21 years. All participants of T1 who had consented to further contact were invited to participate in this follow-up. In all, 570 participants (83% of eligible) completed the follow-up questionnaire, and 550 (80%) completed a diagnostic interview. The current study is based on data from T2.

Out of the 570 adolescents participating in the study, 324 (56.8%) were female and 246 (43.2%) were male. The mean age of the participants was 18.6 years (SD 1.7).

### Interpersonal violence (IPV)

Experiences of IPV were conceptualized as exposure to physical, psychological or sexual violence within the family, an intimate partner relationship or the community in line with the World health organization conceptualization of violence, including child maltreatment and bullying [31]. The questionnaire in the CAP survey included several questions on events of potentially traumatic character, of which five items were categorized as IPV.

The participants were asked whether they have encountered any of the following events: (1) being exposed to violence (to be beaten up or hurt), (2) witnessing others being exposed to violence, (3) being exposed to unpleasant sexual acts by peers, (4) being exposed to unpleasant sexual acts by adults, and (5) being threatened or physically harassed by someone for a longer time period (bullying). The alternative answers were “No”, “Yes, over the past year” and “Yes, over the past 3 years”, which were dichotomized into “No, not experienced” and “Yes, over the past 3 years”.

### Recurrent headache

The CAP survey also included questions about frequent pain, phrased as: “How often have you experienced the following complaints over the past 3 months, without having hurt yourself or having a known disease causing the pain?” The adolescents reported pain localization, including headache or migraine, and frequency ranging from “Never/rarely”, “Approximately once a month”, “Approximately once a week”, “Several times a week” and “Almost every day”. “Approximately once a week”, and “Several times a week” were collapsed to one category and responses were categorized as “Never/rarely”, “Monthly”, “Weekly” or “Daily” headaches.

### Psychiatric disorders

At T1 main psychiatric diagnoses were registered for the participants, according to the International Classification of Diseases and Related Health Problems, 10th edition (ICD-10) [32]. Data from the baseline study show that hyperkinetic disorders were the most common disorders in the population (38.2%), while 26.2% fulfilled diagnostic criteria for anxiety disorders, 15.4% for mood disorders, 6.9% for autism spectrum disorders, and 3.9% for eating disorders [1]. At T2 a semi-structured diagnostic interview was conducted using the Schedule for Affective Disorders and Schizophrenia for School-Age Children (K-SADS) to assess psychiatric disorders [33]. The interviewers all had a graduate degree in medicine or psychology, and had clinical experience from child and adolescent psychiatric health services.

### Statistics

Descriptive data were analyzed using the Pearson Chi-Square test. The risk of recurrent headache according to the number of types of IPV was analyzed using binary logistic regression, adjusting for sex and age. An IPV exposure sum-score was created based on respondents’ exposure to the five types of IPV measured in the study. Due to low numbers of scores 3–5, these were combined into one category (≥ 3), resulting in a range from 0–1–2– ≥ 3. *P* < 0.05 were considered statistically significant. The statistical analyses were performed using IBM SPSS Statistics v.25 and v.27 (IBM Corp., Armonk, NY).

## Results

Sex differences in headache frequency and exposure to IPV are displayed in Tables 1 and 2. Overall, about a third of the adolescents reported frequent headaches (weekly or daily). Almost every second girl reported frequent headaches, while one in five boys experienced such complaints. Table 1 also shows that among participants with frequent headaches two-thirds were diagnosed with a psychiatric disorder. Thus, adolescents with more severe mental problems were more likely to experience frequent headaches as compared to their peers who did not fulfill criteria for a psychiatric disorder.

**Table 1:** Headache frequency by age, sex and present psychiatric disorder

	All		Headache frequency				*P* value
			Never/rarely	Monthly	Weekly	Daily	
	N	Mean (SD)/(%)	Mean (SD)/*N* (%)	Mean (SD)/*N* (%)	Mean (SD)/*N* (%)	Mean (SD)/*N* (%)	
Age, years	570	18.6 (1.7)	18.3 (1.6)	18.9 (1.6)	18.8 (1.7)	19.4 (1.6)	
Gender							< 0.001
Male	246	43.1	147 (60.7)	47 (19.4)	45 (18.6)	3 (1.2)	
Female	324	56.8	86 (26.7)	88 (27.3)	121 (37.6)	27 (8.4)	
Psychiatric disorder	541						0.003
Yes	296	54.7	108/226 (47.8)	65/126 (51.6)	101/155 (65.2)	19/28 (67.9)	
No	245	45.3	118/226 (52.2)	61/126 (48.4)	54/155 (34.8)	9/28 (32.1)

**Table 2:** Exposure to types of interpersonal violence by gender and age

Interpersonal violence type	Age mean (SD)	Female *N* (%)	Male *N* (%)	*P* value
Physical violence	19.0 (1.7)	60 (18.5)	38 (15.7)	0.381
Witnessing violence	19.0 (1.7)	65 (20.1)	66 (27.3)	0.044
Unpleasant sexual acts by peers	19.2 (1.7)	61 (18.9)	4 (1.7)	< 0.001
Unpleasant sexual acts by adults	19.1 (1.7)	31 (9.6)	5 (2.1)	< 0.001
Bullying	19.2 (1.6)	50 (15.5)	22 (9.1)	0.025

As expected, exposure to interpersonal violence was unequally distributed among the sexes (Table 2). Overall, about one in five girls and boys reported exposure to physical violence. Boys were more prone to experience witnessing violence, while girls reported exposure to unpleasant sexual acts by peers or adults 5–10 times more frequently than boys. Exposure to bullying was also more frequently reported by girls as opposed to their male counterparts.

Adolescents exposed to unpleasant sexual acts or bullying reported more frequent headaches (weekly and daily) as opposed to their peers, not exposed to the particular type of IPV (Table 3). Differences between groups were, however, not significant for exposure to unpleasant sexual acts from adults, possibly due to low numbers. In addition, there seemed to be a trend of more frequent headaches among adolescents exposed to physical violence, although not statistically significant (*p* = 0.070). Witnessing violence was not related to higher frequency of headaches. Overall, about 40% of the adolescents reported exposure to any interpersonal violence, and about one in ten had experienced three or more types of violence (Table 4). Participants exposed to three or more types of IPV seemed to be at particularly high risk of experiencing frequent headache.

**Table 3:** Exposure to interpersonal violence by frequency of recurrent headache

Interpersonal violence type		Recurrent headache				*P* value
		Never/rarely *N* (%)	Monthly *N* (%)	Weekly *N* (%)	Daily *N* (%)	
Physical violence	Exposed	30 (30.6)	24 (24.5)	36 (36.7)	8 (8.2)	0.070
	Non-exposed	200 (43.3)	111 (24.0)	129 (27.9)	22 (4.8)	
Witnessing violence	Exposed	45 (35.2)	35 (27.3)	40 (31.3)	8 (6.3)	0.464
	Non-exposed	185 (42.8)	100 (23.1)	125 (28.9)	22 (5.1)	
Unpleasant sexual acts by peers	Exposed	9 (14.1)	18 (28.1)	29 (45.3)	8 (12.5)	< 0.001
	Non-exposed	219 (44.3)	117 (23.7)	136 (27.5)	22 (4.5)	
Unpleasant sexual acts by adults	Exposed	9 (25.7)	8 (22.9)	15 (42.9)	3 (8.6)	0.157
	Non-exposed	221 (42.3)	126 (24.1)	149 (28.5)	27 (5.2)	
Bullying	Exposed	14 (19.4)	19 (26.4)	30 (41.7)	9 (12.5)	< 0.001
	Non-exposed	215 (44.2)	116 (23.9)	134 (27.6)	21 (4.3)

**Table 4:** Recurrent headache and exposure to number of types of interpersonal violence

	*N* (%)	B	Odds ratio (95% CI)	*P* value
IPV sum score				0.014
0	346 (61.8)			
1	113 (20.2)	−0.036	0.964 (0.599, 1.551)	0.881
2	51 (9.1)	0.084	1.087 (0.571, 2.070)	0.799
≥ 3	50 (8.9)	1.040	2.829 (1.484, 5.393)	0.002
Sex		−1.150	0.317 (0.213, 0.471)	< 0.001
Age		0.060	1.062 (0.948, 1.189)	0.300

## Discussion

The present findings demonstrated that adolescents and young adults in the mental health services commonly experience frequent headaches in addition to the mental health problems they were referred for. Adolescents with more severe mental health problems had more frequent headaches than their peers not fulfilling diagnostic criteria for psychiatric illness. Exposure to sexual abuse, bullying and multiple types of interpersonal violence seemed to increase risk of frequent headaches in this clinical population of adolescents. These findings suggest that frequent headaches should be included as part of a need-based approach to better help adolescent psychiatric patients. Exposure to interpersonal violence may impact not only on the psychiatric symptomatology, but also on the pain of adolescents.

Previous research results published from the current study, has reported that many struggle with substantial lifestyle challenges and risky health behaviors, such as smoking and trying illicit drugs [34], social anxiety [35], low levels of physical activity [36] and being victims of bullying [6].

This study demonstrates that frequent headaches are common in this clinical population of adolescents and young adults with a history of mental health problems. This is a higher prevalence than what is reported in studies on the general population [18]. Frequent headache weekly or more often is clinically relevant and is likely to affect daily life function of these individuals. Early pain and somatic symptoms after severe traumatic events, have been shown to predict later psychopathology [5]. In the baseline study of the CAP survey, 70% of the adolescents reported chronic pain, and 41% reported chronic headache or migraine (pain at least weekly over the past 3 months) [1]. These results indicate that the prevalence of headache in the CAP survey population has been relatively stable from baseline study at ages 13–18 years, to follow-up study at ages 16–21 years.

For IPV there were differences in prevalence, with girls reporting significantly higher exposure to sexual abuse by both peers and adults, and being bullied than boys, and with boys reporting witnessing violence more often than girls. Sexual abuse being more common in females is consistent with previous research findings [37, 38]. For being threatened or physically harassed, the research findings vary. A Norwegian study on adolescents (aged 10–16 years) found no significant gender difference for being a victim of school bullying [39], and another Norwegian study on adolescents (aged 12–15 years) found no significant gender difference for teasing and exclusion, but that boys were reporting significantly more physical assault than girls [40]. A cross-national review of self-report studies for ages 11, 13 and 15 years in 40 countries found that girls were victims of bullying more often than boys in 29 out of the 40 countries [41], which complies with the findings in this study. For witnessing parental violence, one study on a Norwegian general population sample found no gender difference in prevalence [38], which differs from the results of this study.

Looking at different types of IPV, we found an association with recurrent headache only for sexual abuse by peers and bullying. For the other types of IPV, there were no statistically significant relationships. These results differ from the findings of the Young-HUNT 3 study, which investigated the same types of IPV as the CAP survey in the adolescent general population in Norway, where the relationship with frequent headache was consistent for all types of IPV for girls, and for all types except for sexual abuse by peers or adults for boys [42].

This study demonstrates that experiences of IPV are highly relevant when studying recurrent headache in a clinical population of adolescents and young adults with a history of psychiatric problems. The risk of recurrent headache does not increase significantly with an increasing number of IPV experienced until reporting three or more types of IPV. This is not consistent with previous research on this relationship in the general population, which demonstrate a dose–response association [18, 27–29], but it demonstrates the same tendency. However, it supports the hypothesis that there is an increased risk of recurrent headache in clinical psychiatric populations. Exposure to several types of IPV, that are relatively frequent in this population, further increases the risk of recurrent headache. The higher prevalence of headache in this specific population could, therefore, be a consequence of the underlying psychiatric disorder, and not only of exposure to IPV.

Many studies have demonstrated an association between psychiatric disorders and pain. For instance, a study on a Canadian general population sample aged 15 years and older found that major depressive disorder, bipolar disorder, panic disorder and social phobia were all twice as prevalent in those with migraine compared with non-migraineurs [43]. Furthermore, mental problems seem to persist to a higher degree in the presence of chronic headache and pain.

There are several plausible, partially overlapping mechanisms that could inform what role headaches may have in relation to mental health problems, and how such pain may hinder recovery. Mechanisms could relate to headaches (a) leading to excessive functional impairment, (b) functioning as potent reminders of threat, (c) reflect more extensive underlying neurobiological alterations (d) impact access to or efficiency of early interventions [5]. A review on neuroimaging studies suggested that the association between migraine and psychiatric disorders could be a result of dysfunction of the pathways modulating pain in the limbic system and the brainstem. This would comply with a neurolimbic migraine model, where in addition to central sensitization in the brainstem, the model also comprises limbic dysfunction and cortical hyperexcitability. Coping strategies, emotions, mood, personality and stress are all factors of the limbic system, and factors such as these can trigger migraine and lead to increased susceptibility to migraine attacks. Limbic functions are again influenced by the dysfunction of the brainstem, indicating bidirectionality of the influence [44]. Therefore, limbic dysfunction could serve as a link between migraine and psychiatric disorders.

### Implications for clinicians

The results of the current study shows that headache is a common comorbid disorder among adolescents in child and adolescent psychiatric care, and even more common among patients with IPV experiences. The study underlines the importance of a broad assessment of children and adolescents in CAP services, including pain disorders. The combination of psychiatric symptoms and pain could indicate severe underlying factors, such as previous or ongoing IPV experiences. Current knowledge show promising results from cognitive or narrative therapy in alleviating the effects that traumatic experiences and pain can have on the body [45]. Headache and other somatic complaints in the early phase after trauma exposure seems to increase the risk of persistent symptoms [5]. Early identification of children and adolescents with high risk of developing chronic psychiatric disorders could be of high importance to improve prognosis and treatment outcome. For the clinician, it is, therefore, important to address symptoms of headache in addition to psychiatric symptoms to improve treatment outcome for the individual.

### Strengths and limitations

One of the strengths of this study is the large sample from a clinical population. The data material is unique, as it is a follow-up study of a clinical child and adolescent psychiatry unit population.

Another strength of this study is that the number of participants completing the follow-up was high, with 83% of the participants from T1 responding to the follow-up questionnaire and 80% completing a diagnostic interview.

The history of experiences of IPV and headache was based on retrospective self-report for 3 years and 3 months prior to the time of response, respectively. In such studies, the reports could be influenced by recall bias. Reports of IPV could also be uncertain due to the psychological reactions after traumatizing experiences, which may affect the memory process; and the stigma of being an abuse victim could give response bias. However, in this study population consisting of adolescents and young adults, the time passed since potentially having these experiences is considerably shorter than in self-report studies on adult populations. This, as well as the shorter time periods asked to report from (3 months for headache and 3 years for IPV), contributes to a reduction of recall bias in our study compared to previous studies on adult populations, which examines for lifetime experiences of IPV in childhood or adolescence. However, the prevalence of reported exposure to sexual abuse by males was low, at 1.7% (*n* = 4) for sexual abuse by peers and 2.1% (*n* = 5) for sexual abuse by adults. This makes the analyses of associations with frequent headaches unreliable in this group. We also lack more specific information on the interpersonal events, such as severity, frequency, relation to the offender, and how recently and for how long the events occurred. These are factors which possibly could affect the strength of the relationship. In addition, as we focused on IPV, we did not assess all types of childhood maltreatment which may contribute to an association with headache, e.g., physical and emotional neglect.

Another limitation of this study is that the definition of ‘frequent headache’ in the questionnaire could be more precise, for instance by defining the headache characteristics by the diagnostic criteria of the International Classification of Headache Disorders II [46]. The respondents have not completed specific headache interviews to further divide into the different subtypes of headache.

The current study is cross-sectional as we focused on data from one timepoint. As a consequence, we cannot conclude about causality from these data. For determining whether a cause–effect relationship exists, there is a need for longitudinal studies. The CAP survey is a longitudinal study, and future research comparing the data from the current study with data from other follow-ups of the CAP survey is a natural next step in the investigation of the relationship between IPV and frequent headaches in this population.

## Conclusions

The main findings of this study are (i) the high prevalence of frequent headaches among adolescents with a history of CAP service use, particularly evident among those with more severe psychiatric symptomatology and (ii) the relationship between exposure to IPV in childhood and adolescence, and later frequent headache among adolescents and young adults with a history of psychiatric problems. Working in CAP units, it is of great importance to be aware of IPV as an important risk factor for both frequent headaches and psychiatric disorders. In clinical practice, pain conditions such as headache should be evaluated, and in all patients exposure to IPV should be assessed. Knowledge of adolescents’ psychiatric symptomatology, pain and headaches and their history of exposure to interpersonal violence and other trauma should guide the clinician to provide improved need-based and trauma-informed health services to this vulnerable group of patients, and thereby possibly increase chance of recovery and reduce the risk of chronification of pain disorder and development of comorbid psychopathology and psychosocial problems.

It is likely that early intervention could prevent development of chronic headache and poor health, and thereby improve the future health of these individuals [47, 48]. If clinicians are able to detect this pain condition, while the patients still are at a young age, it could provide an opportunity to initiate interventions or treatment that could prevent the development of a chronic headache disorder, potentially resulting in poor quality of life, and if the pain condition can be treated, there is an improved chance of recovery from mental health problems [5].

## Data Availability

The data sets used and/or analyzed during the current study are available from the corresponding author upon reasonable request.
